# Predictors for hospital admission in emergency department patients with benign paroxysmal positional vertigo: A retrospective review

**DOI:** 10.1371/journal.pone.0280903

**Published:** 2023-01-24

**Authors:** Jennifer Rizk, Moustafa Al Hariri, Malak Khalifeh, Abdo Mghames, Eveline Hitti

**Affiliations:** 1 Department of Emergency Medicine, American University of Beirut Medical Center, Beirut, Lebanon; 2 Vice President for Medical and Health Sciences, QU Health, Qatar University, Doha, Qatar; Sapienza University of Rome, ITALY

## Abstract

**Objective:**

This study aims to assess the incidence of Emergency Department (ED) visits for benign paroxysmal positional vertigo (BPPV), describe patient characteristics, management practices and predictors of inpatient admission of BPPV patients.

**Methods:**

This was a retrospective chart review of patients presenting with BPPV to a single ED between November 2018 and August 2020. Patients’ characteristics, ED management, discharge medications, disposition and unscheduled return visits were determined.

**Results:**

In total, 557 patients were included. Average age was 49 years, 54.2% were females and 12.4% required hospital admission. In the ED, 51.1% received intravenous hydration, 33.8% received anti-emetics, 10.1% received benzodiazepines, 31.8% underwent canalith repositioning maneuvers (CRMs) and 56.7% were discharged on acetyl-leucine. Of discharged patients, 2.5% had unscheduled return visits. A higher likelihood of admission was associated with age above 54 years (aOR = 4.86, p<0.001, 95% CI [2.67, 8.86]), home use of proton pump inhibitors (PPIs) (aOR = 2.44, p = 0.03, 95% CI [1.08, 5.53]), use of anti-emetics and benzodiazepines in the ED (aOR = 2.34, p = 0.003, 95% CI [1.34, 4.07]) and (aOR = 2.18, p = 0.04, 95% CI [1.03, 4.64]), respectively.

**Conclusion:**

While BPPV is a benign diagnosis, a significant number of patients presenting to the ED require admission. Predictors of admission include older age, PPIs use and ED treatment with anti-emetics and benzodiazepines. Although CRMs are the gold standard for management, CRMs usage did not emerge as protective from admission, and our overall usage was low.

## Introduction

Dizziness is a common complaint for Emergency Department (ED) presentations representing 3 to 4% of ED volumes [1, 2]. Dizziness could indicate a wide range of disorders from benign, yet disruptive ear problems such as vertigo, to serious neurological problems such as stroke. Vertigo is characterized by frequent, irritating and curable symptoms that could have central or peripheral causes with peripheral ones accounting for around 60% of all vertigo cases [2, 3]. While vestibular migraine is the most common cause of episodic vertigo [4], benign paroxysmal positional vertigo (BPPV) is the primary cause of peripheral vertigo with a lifetime prevalence of 36%, lifetime incidence of 2.4% and an annual incidence of 0.6% [1, 5, 6].

BPPV is characterized by an acute, episodic and rotational sensation caused by the displacement of otoconia into the semicircular canals [1, 7]. BPPV can involve the anterior, horizontal or posterior canals of the inner ear. Each type of BPPV has its own diagnostic test, however, posterior canal BPPV is considered the most common in patients with BPPV and can be diagnosed using Dix-Hallpike test (DHT) [8]. DHT is a simple bedside test effective for the diagnosis of BPPV by provoking torsional and vertical eye nystagmus. Canalith Repositioning Maneuvers (CRMs) have strong evidence for their effectiveness in treating BPPV [9, 10].

ED management practices for BPPV however are variable. For instance, a study conducted on patients selected from EDs in Texas showed a decrease in DHT utilization in patients presenting with dizziness and vertigo [11]. Additionally, Bhattcharayya et al. showed that Computed Tomography scans (CT scans) and Magnetic Resonance Imagings (MRIs) are still used in more than one third of the BPPV patients to rule out central causes of vertigo, despite the guidelines’ recommendations against the use of such procedures [12]. Most studies focus on the diagnosis and management of BPPV but to our knowledge, no study has assessed predictors for hospital admission in BPPV patients presenting to the ED.

In Lebanon, there is a paucity of data on BPPV presentations to EDs and their clinical management. Moreover, the clinical characteristics of BPPV patients and their ED disposition were not previously evaluated. In the current study, we aim to assess the incidence rate of BPPV presentations, describe their clinical characteristics, their management practices, and assess the predictors for hospital admission in a single-center ED at a tertiary care center in Lebanon.

## Methods

### Study design

This was a single-center retrospective chart review study conducted in an academic ED at the American University of Beirut Medical Center (AUBMC). AUBMC is a large tertiary care center in Lebanon. It has 358 beds and receives approximately 55,000 ED visits annually with a 20% admission rate. Approval for the present study was granted by the Institutional Review Board at AUBMC under the protocol ID BIO-2019-0039.

### Selection of participants

This study was conducted on 557 adult (≥ 18 years old) patients who presented to the ED between January 2018 and August 2020, with a chief complaint of dizziness/vertigo and a discharge diagnosis of BPPV. The patients’ ED encounters were filtered by an experienced data user using the hospital’s electronic health record system via structured keyword searches and ICD-10 coding, (International Statistical Classification of Diseases 10^th^ Revision and Related Health Problems). The electronic medical records and ED notes were then accessed and reviewed to exclude patients with an alternative final diagnosis. This included patients with consultation notes and imaging results providing alternative diagnoses as well as patients who were admitted and then discharged with a diagnosis other than BPPV.

### Data collection

Our methods for data collection adhere to the criteria suggested by Worster et al. for retrospective chart reviews [13]. Two research fellows were trained to review medical charts retrospectively in order to collect our variables of ft. General demographics and baseline characteristics of BPPV patients including third party payer, smoking status, alcohol use, medical family history, past medical history comprising history of BPPV and other ear problems, comorbidities and home medications were collected. Clinical characteristics were also retrieved and included patient presentation and chief complaint, DHT positivity, emergency severity index (ESI), duration and severity of BPPV episode and systolic blood pressure of BPPV patients. Our data collection also included ED management of BPPV patients, imaging studies conducted and whether or not an Otolaryngologist (ENT) or Neurology specialist had been consulted in the ED. Discharge medications and unscheduled return visits were also extracted from the database.

### Statistical analysis

Statistical analysis was performed using SPSS version 25.0 (Armonk, NY: IBM Corp). The distributions of the continuous variables were presented as mean ± standard deviation and categorical variables as frequency and percentages. Selected characteristics were then stratified by admission status (discharged or admitted) and variable differences between the two groups were calculated by Pearson’s Chi-square or Fisher’s exact test and Student’s *t* test, where appropriate. ROC curve was performed to determine the cut-off point for age as a predictor for hospital admission with highest sensitivity and specificity. Tests were interpreted at a significance level alpha = 0.05. A logistic regression was then performed to adjust for confounding variables and to determine the risk factors associated with hospital admission.

## Results

### Baseline characteristics of BPPV patients

A total of 557 patients diagnosed with BPPV were enrolled in this study. The average age was 49 years (± 17.1) and about half were females (54.2%). Hypertension was the highest comorbid condition (30%) and 35.4% of our study population had more than two comorbidities. Antihypertensives (20%) and PPIs (6.3%) were the most common home medications.

The majority of our study population was discharged from the ED (87.6%) and only 12.4% of patients were admitted. Admitted patients to the hospital were significantly older than those discharged from the ED (61.9±15.9 vs 46.7±16.4, p<0.001). Moreover, patients admitted to the hospital had significantly more family history of cardiovascular diseases (CVDs) (27.5% vs 12.1%, p = 0.001) and more comorbidities than discharged patients. Additionally, admitted patients had significantly higher rates of proton pump inhibitors (PPIs) usage than discharged patients (17.4% vs 4.7%, p<0.001) (Table 1).

**Table 1:** Baseline characteristics of BPPV patients.

		Total	Discharged	Admitted	p-value
		N = 557 (%)	n = 488 (%)	n = 69 (%)	
**Age (years)**	**Mean ± SD**	48.7 ± 17.1	46.8 ± 16.4	61.9 ± 15.9	<0.001
**Age (years)**	**≤ 54**	343 (61.6%)	324 (66.4%)	19 (27.5%)	<0.001
	**>54**	214 (38.4%)	164 (33.6%)	50 (72.5%)	
**Sex**	**Female**	303 (54.2%)	260 (53.3%)	43 (62.3%)	0.15
	**Male**	254 (45.8%)	228 (46.7%)	26 (37.7%)	
**Marital status**	**Married**	413 (74.1%)	359 (73.6%)	54 (78.3%)	0.38
	**Others**	144 (25.9%)	129 (26.4%)	15 (21.7%)	
**Third party payer**		496 (89%)	440 (90.2%)	56 (81.2%)	0.01
**Smoker**		146 (26.2%)	126 (25.8%)	20 (29%)	0.6
**Alcohol use**		49 (8.8%)	45 (9.2%)	4 (5.8%)	0.34
**History of BPPV**		61 (11%)	53 (10.9%)	8 (11.6%)	0.87
**Other ear problems**		15 (2.7%)	10 (2%)	5 (7.2%)	0.03
**Family history**	**Cardiovascular diseases**	78 (14%)	59 (12.1%)	19 (27.5%)	0.001
	**Hypertension**	123 (22.1%)	106 (21.7%)	17 (24.6%)	0.6
	**Dyslipidemia**	51 (9.2%)	42 (8.6%)	9 (13%)	0.24
	**Diabetes mellitus**	103 (18.5%)	85 (17.4%)	18 (26.1%)	0.09
**Past medical history**	**Hypertension**	166 (30%)	134 (27.6%)	32 (46.4%)	0.001
	**Dyslipidemia**	107 (19.3%)	86 (17.7%)	21 (30.4%)	0.01
	**Cerebrovascular diseases**	15 (2.7%)	11 (2.3%)	4 (5.8%)	0.1
	**Cardiovascular diseases**	51 (9.2%)	38 (7.8%)	13 (18.8%)	0.003
	**Diabetes mellitus**	78 (14.1%)	62 (12.8%)	16 (23.2%)	0.02
	**Thyroid dysfunction**	33 (6%)	27 (5.6%)	6 (8.7%)	0.28
	**History of dizziness BPPV or vertigo**	102 (18.4%)	85 (17.5%)	17 (24.6%)	0.15
	**Others**	83 (15%)	68 (14%)	15 (21.7%)	0.09
**Number of comorbidities**	**<2**	358 (46.4%)	326 (67.2%)	32 (46.4%)	0.001
	**≥ 2**	196 (35.4%)	159 (32.8%)	37 (53.6%)	
**Home medications**	**Proton pump inhibitors**	35 (6.3%)	23 (4.7%)	12 (17.4%)	<0.001
	**Antihypertensives**	111 (20%)	94 (19.4%)	17 (24.6%)	0.31
	**Anxiolytics and sedatives**	30 (5.4%)	26 (5.4%)	4 (3.7%)	0.78
	**Alpha-blockers**	5 (0.9%)	4 (0.8%)	1 (1.4%)	0.49
	**Bisphosphonates**	2 (0.4%)	1 (0.2%)	1 (1.4%)	0.23

Data are presented as mean with standard deviation (± SD) or numbers with percentages as appropriate.

p-value for difference between two adjacent columns is calculated by chi-square or Fisher´s exact test or T test, where appropriate.

Abbreviations: BPPV: Benign Paroxysmal Positional Vertigo.

The analysis of the ROC curves performed to determine the cut-off point for age as a predictor for hospital admission showed that the discriminative ability was reasonable for the age with a cut-off value of 54 years (AUC = 0.746, 95% CI: 0.685–0.807, sensitivity = 0.754, 1-specificity = 0.353).

### Clinical characteristics of BPPV patients

Most ED patients presenting with vertigo or dizziness (86.3%), followed by nausea (12.5%), vomiting (9.4%) and headache (3.1%). DHT was reported in 71.6% of the patients and was positive in 71.9% of the cases. The majority had the BPPV episode for less than a week (91.8%) and an ESI of 3 (94.4%).

Admitted patients had significantly more vomiting on initial presentation than patients discharged from ED (17.4% vs 8.2%, p = 0.01). However, no significant difference was found in terms of ESI, DHT positivity, severity of the symptoms, duration of BPPV episode and systolic blood pressure when comparing discharged to admitted patients (Table 2).

**Table 2:** Clinical characteristics of BPPV patients.

		Total	Discharged	Admitted	p-value
		N = 557 (%)	n = 488 (%)	n = 69 (%)	
**Chief complaint and presentation**	**Vertigo or dizziness**	478 (86.3%)	426 (87.8%)	52 (75.4%)	0.005
	**Nausea**	69 (12.5%)	59 (12.2%)	10 (14.5%)	0.58
	**Vomiting**	52 (9.4%)	40 (8.2%)	12 (17.4%)	0.01
	**Headache**	17 (3.1%)	13 (2.7%)	4 (5.8%)	0.25
**Dix-Hallpike test (DHT)**	**Test performed**	399 (71.6%)	348 (71.3%)	51 (73.9%)	0.673
	**Positive test**	287 (71.9%)	253 (72.7%)	34 (66.7%)	0.701
**ESI**	**1–2**	26 (4.7%)	20 (4.1%)	6 (8.7%)	0.15
	**3**	523 (94.4%)	461 (95.1%)	62 (89.9%)	
	**4–5**	5 (0.9%)	4 (0.8%)	1 (1.4%)	
**Duration of BPPV episode**	**<1week**	505 (91.8%)	439 (91.3%)	66 (95.7%)	0.21
	**>1week**	45 (8.2%)	42 (8.7%)	3 (4.3%)	
**Severity**	**Mild**	210 (37.9%)	186 (38.4%)	24 (34.8%)	0.51
	**Moderate**	199 (35.9%)	176 (36.3%)	23 (33.3%)	
	**Severe**	145 (26.2%)	123 (25.4%)	22 (31.9%)	
**Systolic blood pressure (mmHg)**	**Mean (SD)**	133.9 ± 17.7	134.2 ± 17.5	132.4 ± 19.3	0.44

Data are presented as mean with standard deviation (± SD) or numbers with percentages as appropriate.

p-value for difference between two adjacent columns is calculated by chi-square or Fisher´s exact test or t-test, where appropriate.

Abbreviations: ESI: Emergency Severity Index.

### ED management of BPPV patients

More than half the patients (51.1%) received IV hydration whereas 31.8% underwent CRMs in the ED. Anti-emetics (33.8%) and benzodiazepines (10.1%) were the most commonly used medications in the ED. Furthermore, imaging studies were obtained in 32.7% of the patients and ENT /Neurology consults were ordered on 16.8% and 20% respectively.

A greater percentage of patients admitted to the hospital received anti-emetics (53.6% vs 30.9%, p<0.001) and benzodiazepines (18.8% vs 8.9%, p = 0.01) than discharged patients. Admitted patients also had more ENT (30.4% vs 14.8%, p<0.001) and Neurology consults (60.9% vs 14.2%, p<0.001). They also underwent more MRIs (11.6% vs. 0.8%, p<0.001) and CT scans (78.3% vs. 26.2%, p<0.001) than discharged patients (Table 3).

**Table 3:** ED management of BPPV patients.

		Total	Discharged	Admitted	p-value
		N = 557 (%)	n = 488 (%)	n = 69 (%)	
**IV bolus**		283 (51.1%)	216 (44.5%)	67 (97.1%)	<0.001
**CRM**		176 (31.8%)	157 (32.4%)	19 (27.5%)	0.42
**Received ED medications**		213 (38.4%)	170 (35.1%)	43 (62.3%)	<0.001
	**Anti-emetics**	187 (33.8%)	150 (30.9%)	37 (53.6%)	<0.001
	**Benzodiazepines**	56 (10.1%)	43 (8.9%)	13 (18.8%)	0.01
**Imaging**	**CT**	181 (32.7%)	127 (26.2%)	54 (78.3%)	<0.001
	**MRI**	12 (2.2%)	4 (0.8%)	8 (11.6%)	<0.001
**Consults in the ED**	**ENT physician**	93 (16.8%)	72 (14.8%)	21 (30.4%)	0.001
	**Neurology**	111 (20%)	69 (14.2%)	42 (60.9%)	<0.001

Data are presented as numbers with percentages.

p-value for difference between two adjacent columns is calculated by chi-square or Fisher´s exact test where appropriate.

Abbreviations: ESI: Emergency Severity Index, ED: emergency department, IV: Intravenous, ENT: Ears Nose and throat, CT: Computed Tomography.

### Discharge medications and unscheduled return visits of discharged patients

More than half of the discharged patients (56.7%) were prescribed acetyl-leucine as part of their discharge medications and only 2.5% of them had unscheduled return visits to the ED at one-week post discharge (Table 4).

**Table 4:** Discharge medications and unscheduled return visits of discharged patients.

		n = 488 (%)
**Follow-up medications**	**Acetyl-Leucine**	275 (56.7%)
	**Betahistine**	96 (19.8%)
	**Metoclopramide**	79 (16.3%)
	**Ondansetron**	1 (0.2%)
	**Dimenhydrinate**	125 (25.8%)
**Unscheduled return visit** *	**Yes**	12 (2.5%)
	**No**	473 (94.5%)

*Unscheduled return visits at one week post discharge.

Data are presented as numbers with percentages.

### Factors associated with hospital admission

A multivariate logistic regression exploring variables associated with hospital admission is presented in Table 5. Patients older than 54 years old were about 4 times more likely to be admitted to the hospital than patients younger than 54 years old (aOR = 4.86, p<0.001, 95%CI [2.67,8.86]). Furthermore, patients using PPIs as a home medication were more likely to be admitted to the hospital (aOR = 2.44, p = 0.03, 95%CI [1.08, 5.53]). Finally, patients who received anti-emetics (aOR = 2.34, p = 0.003, 95%CI [1.34, 4.07]) and benzodiazepines (aOR = 2.18, p = 0.04, 95%CI [1.03, 4.64]) in the ED were more likely to be admitted.

**Table 5:** Logistic regression: Factors associated with hospital admission.

	Adjusted OR	p value	95% C.I. for adjusted OR	
**Insurance**	0.48	0.05	0.23	1.005
**Cerebrovascular diseases**	3.48	0.06	0.96	12.61
**Proton pump inhibitors**	2.44	0.03	1.08	5.53
**Anti-emetics in ED**	2.34	0.003	1.34	4.07
**Benzodiazepines in ED**	2.18	0.04	1.03	4.64
**Age >54 years**	4.86	<0.001	2.67	8.86

Note: variables entered in the logistic regression model are variables with p-value < 0.2 in bivariate analysis: age (reference: ≤54 years), Sex (reference: Male), Insurance (reference: no), ESI, hypertension (reference: no), dyslipidemia (reference: no), Cerebrovascular disease (reference: no), Cardiovascular disease (reference: no), diabetes mellites (reference: no), History of dizziness or BPPV (reference: no), use of proton pump inhibitors at home (reference: no), Anti-emetics in ED (reference: no), Benzodiazepines in ED (reference: no), number of comorbidities (reference:<2), and Rehab Maneuver (reference: no).

Omnibus test < 0.001, R2 = 0.213, Hosmer = 0.932

Abbreviations: ESI: Emergency Severity Index, BPPV: Benign Paroxysmal Positional Vertigo (BPPV), ED: emergency department, IV: Intravenous, aOR: adjusted odds ratio; 95%CI: 95% confidence interval.

## Discussion

This retrospective chart review study is the first to look at predictors for admission for patients who present to the ED with BPPV. Our results found that more than half of BPPV patients presenting to the ED were female and the majority were married, had DHT performed, an ESI of 3 and presented with acute vertigo symptoms. Most of the patients presenting with BPPV were discharged home. More than half of them received IV hydration and around one third received anti-emetics. Less than a third of the patients received a CRM despite the strong scientific evidence supporting its usefulness in the management of BPPV [8]. When comparing baseline and clinical characteristics between admitted and discharged patients, admitted patients were more likely to be older than 54 years. In addition, admitted patients were more likely to be taking PPIs as part of their home medications, have CVD in their medical history, have no insurance coverage, and receive benzodiazepines and anti-emetics in the ED.

The one-year incidence of BPPV in our study was 0.72%, which is 1.5 times higher than the rate of a single-center study conducted in an ED in the United Sates (US) (0.46%). Even though there has been some association between COVID-19 infection, vaccines and vertigo [14, 15], we do not believe this contributed to the higher incidence in our study as Lebanon was following a high stringency containment strategy during the study period with a test positivity rate of 1.5% and an incidence of 251 cases per million [16]. Moreover, vaccination campaigns in Lebanon had not started yet during the study period. One possible explanation for the higher BPPV incidence rate in our study could be related to a high prevalence of vitamin D deficiency in the Lebanese population which has been associated with BPPV [17, 18]. Emerging data suggests Vitamin D supplementation may reduce the risk of BPPV recurrence [19]. This could explain the higher incidence of BPPV in our study although wider population data is needed to assess this further.

In line with Kerber et al., our results showed that a higher portion of patients who present to the ED with BPPV are females [11]. Moreover, the cut-off value for age as a predictor for hospital admission derived from the ROC curve of our study participants was 54 years old, which is comparable to Kerber et al. study done in Texas [11] (60.2 years old) and Koelliker et al. study done in Mississippi (50 years old) [20].

The majority of patients presenting with BPPV in our study were discharged home. This is within the range reported in the literature that includes a high of 24% in Australia [3] and a low of 6% in Berlin [6]. The multivariable analysis showed that patients older than 54 years old were about five times more likely to be admitted to the hospital. However, we cannot conclude whether this is a causality relationship or whether physicians are more likely to admit older symptomatic patients who might have central etiologies for their acute symptoms. Another predictor for hospital admission was the use of PPIs as part of the home medications. In fact, 17.4% of our admitted patients were on PPIs compared to 4.7% of the discharged patients. Messina et al. showed that 28% of their study population with BPPV diagnosis were taking PPIs [21]. Picciotti et al. also demonstrated that patients on PPIs had more recurrence of BPPV symptoms than those who were not on PPIs [22]. The main component of otoconia is known to be calcium carbonate. Gastric acid suppression leads to hypochlorhydria which interferes with calcium absorption in its water insoluble form (calcium carbonate). This could explain the associated risk between PPIs use and otoconial decalcification resulting in BPPV.

CRMs are considered the gold standard treatment for BPPV. In our study 38.1% of our patients were treated with CRMs, a much higher percentage than that shown in other studies where CRMs usage ranged between 3.9% and 7% [11, 23]. Although CRMs did not emerge as a protective factor for admission in our multivariate regression, our study design did not aim at exploring this question from a causality perspective.

While benzodiazepines are not part of the guidelines for BPPV management [24–26], their use has been shown to be effective for symptoms relief of both vertigo-related and non-vertiginous dizziness [27]. In our study, patients who received benzodiazepines in the ED were two times more likely to be admitted. This is likely more reflective of the severity of presenting symptoms prompting physicians to administer symptomatic treatments rather than a causality relationship, however our study design is unable to make this distinction.

Almost half of the patients in a study conducted by in Italy had recurrent symptoms within few months of their initial presentation, whereas our study showed a recurrence rate of 12.5% in one year and a return visit rate at one week of 2.7%. This could be related to our higher rates of CRMs usage or more accessible outpatient follow-up. Another explanation that deserves further exploration is the use of acetyl-leucine as part of the discharge medications [21]. More than half of our discharged patients (56.7%) were prescribed acetyl-leucine after their discharge from the ED. The use of acetyl-leucine is based on animal studies that showed it enhances all compensatory mechanisms thereby reducing symptoms of dizziness. Given the limited data in human studies, acetyl-leucine usage for BPPV is not wide-spread. While the safety profile of acetyl-leucine includes minimal side effects, further human studies are required to assess its efficacy and safety in BPPV patients [28].

Based on the findings of this study, CRMs usage was the highest yet reported in an ED setting but remains lower than expected given their proven efficacy in aborting BPPV. This suggests the need for more education on CRMs usage in the ED. Also, it is important to recognize that studies exploring effectiveness of CRMs have all been conducted in an ambulatory care settings. How well this data can be extrapolated to an ED setting where patients may be presenting with more severe symptoms remains unclear.

## Limitations

As a retrospective chart review study, our study cannot provide explanations of causality. Furthermore, the data captured on diagnosis and interventions is limited to what is documented in the chart and may not reflect what was actually delivered in the ED. Specifically, even though our rates of CRMs usage are higher than those reported in other studies, such interventions that do not require documented orders may be under-reported. Also, our study was conducted at a single center in Lebanon which could affect external generalizability of our findings. Furthermore, selected patients were the ones with a discharge diagnosis of BPPV only and not those who were assigned the broader diagnosis of vertigo, some of whom could have had BPPV. This could have led to an underestimation of the true incidence of cases in our population.

## Conclusion

While BPPV is a benign diagnosis, a significant number of patients presenting to the ED require admission. Predictors of hospital admission include older age, PPIs use as part of the home medications and treatment with anti-emetics and benzodiazepines in the ED. Although CRMs did not emerge as a protective measure for hospital admission, their overall use in the ED setting remains low. More studies are needed to explore the association between BPPV and PPIs usage and to identify any potential role for acetyl-leucine in the outpatient management of BPPV.

## Supporting information

S1 File (SAV)
